# Food insecurity and eating habits of Lebanese children aged 5–11 years during the COVID-19 pandemic and the socioeconomic crisis: a national study

**DOI:** 10.1186/s12889-022-14387-z

**Published:** 2022-10-29

**Authors:** Reine Gedeon, Souheil Hallit, Lara Hanna Wakim

**Affiliations:** 1 Department of Nutrition, Faculty of Arts and Sciences, Holy Spirit University of Kaslik, P.O. Box 446, Jounieh, Lebanon; 2 School of Medicine and Medical Sciences, Holy Spirit University of Kaslik, P.O. Box 446, Jounieh, Lebanon; 3 Research Department, Psychiatric Hospital of the Cross, Jal Eddib, Lebanon

**Keywords:** Food insecurity, Children, COVID-19 pandemic, Economic crisis, Lebanon

## Abstract

**Background:**

Food insecurity is the lack of access to nutritious healthy food due to economic and financial insufficiencies. Food insecurity is expected to be higher during these difficult times in Lebanon, which is facing many financial, political, economic and health debates. The present study aims to find the prevalence of food insecurity among Lebanese children during the COVID-19 pandemic and its correlates.

**Methods:**

This cross-sectional study enrolled 4001 participants from all Lebanese governorates (March–April 2022). The Ministry of Education and Higher Education randomly disseminated the link to parents of children aged between 5 and 11 years from public and private schools.

**Results:**

The results showed that 1505 (37.6%) and 1497 (37.4%) had moderate and severe food insecurity. A significantly higher percentage of families with severe food insecurity was reported by fathers compared to mothers. In addition, participants who reported a bad overall health status of their children had a severe food insecurity. Moreover, those with a severe food insecurity had their children’s daily snacking habit between meals decreased, with a decreased quantity of meals, intake of vegetables/fruits, the intake of balanced diet, junk food, sugar-sweetened beverages, consumption of sweets/candies/chocolate, consumption of unhealthy food, intake of immunity-boosting food, intake of nutrition supplements, participation in the house chores, number of sleeping hours and sleep quality, as well as stress/anxiety decreased. Finally, a higher mean financial burden was seen in families with severe food insecurity compared to the other groups.

**Conclusion:**

The current study found a high prevalence of moderate to severe food insecurity among Lebanese children during the COVID-19 pandemic. Food insecurity should be seriously discussed in Lebanon due to its rapid development in the middle of all the crises facing the country in order to avoid short and long term consequences on human’s health.

## Background

The Coronavirus Infectious Disease was first detected in Wuhan, China on December 31, 2019 [1]. The World Health Organization (WHO) declared it as a pandemic on March 11, 2020 [2]. To decrease the outbreak, the WHO recommended several lockdowns. This decision has threatened the well-being, physical and psychological health of the whole population. Social measures to reduce the coronavirus outbreak included partial and total lockdowns that led to many consequences especially in younger population. Children were more susceptible to be affected by the lockdowns; they developed many emotional instabilities including fear, nervousness, mood changes, eating disorders and sleep disorders [3, 4]. Closure of schools for children with all the social restrictions, increased the risk of mental disorders, depression, and anxiety along with lifestyle changes due to lockdown like decreased physical activity, unhealthy diet, increased screen exposure, loneliness, sleep disorders and dealing with the circumstances of their parents’ financial issues triggered by the lockdown [5]. Even though the closure of stores could be beneficial for the decrease in the availability of fast food and for having more time to cook at home [6], nevertheless the lack of fresh food led to an increase in the consumption of comfort sugar-rich food [3]. Unhealthy food in children can lead to chronic health troubles such as obesity, hypertension, cardiac disease, osteoporosis and high risk of cancer with long term health impact later in their life [7]. Reversing children’s bad eating habits acquired during the lockdowns are recently a real concern of pediatricians [8].

Although the pandemic’s burden was global, the consequences differed between nations [9]. Low-to-middle-income countries with fragile economic infrastructures, wars and humanitarian crises were the most affected negatively by the pandemic (such as Arab countries, Latin America and the Caribbean), with an approximate estimate fall of 9.1% in the regional gross domestic product in 2020 because of the pandemic [10]. FI is another problem that adds to the pandemic, with around 2 billion people suffering from FI worldwide [11]. In 2020, 8.3 million and 2 million people in the Arab region were anticipated to fall into poverty due to the pandemic and be food insecure respectively [12].

The impact of COVID has been worse in Lebanon, a low-middle income country in the Middle East, currently facing economic, political and financial crises with one of the largest blasts in the world hit its capital Beirut on August 4, 2020. Constraints on movement, the instability of food prices and interruption of food supply chains, endangered food security of population groups worldwide [13, 14]. Lebanon recorded its first COVID-19 case on February 21, 2020 [15] and faced difficulties enduring the lockdown because of the massive unprecedented crises. This can lead to expected increase in child malnutrition, decreased availability and affordability of nutritious food [16] due to lower income and unemployment. In addition, the large number of Syrian refugees added further taxes on the very restricted economic and financial resources of the country since many years from now, making food insecurity even higher in the Lebanese population [17]. Food insecurity has been a serious global challenge and was one of the most dangerous outcomes of the pandemic; a sample of 378 Lebanese households showed that 50% of mothers coming from a food insecure household were more prone to suffer from unhealthy, inadequate and undesired diet with an increased risk of obesity which can negatively affect their own health and their children [18].

By definition, food insecurity is the lack of access to nutritious healthy food due to economic and financial insufficiencies [19]. A relatively high level of food insecurity was found in around 7 billion a year preceding the pandemic [20], with this number having been expected to increase significantly during the COVID-19 outbreak [21, 22]. The impact of COVID on jobs interrupted the food supply because of many unemployment, thus people tended to purchase affordable food; chasing food price over food quality [23]. School closures deprived around 30 million children of free meals for lunch and exposed them to food insecurity [24] and higher snacking for the lower cost of snacks [25]. Quality of food deteriorated because of lower family financial incomes, with a decrease in consumption of fruits, grains, seafood and vegetables [26, 27]. Study reports during the pandemic showed higher consumption of chips, sugary drinks, red meat and less healthy food [25, 28]. Higher rates of food insecurity were also found in lower-income people, non-Hispanic whites, females and those with lower education level [29–32].

The worsening of the economic status in the Lebanese population led to a reduction in the Lebanese monthly income and a shortage in food affordability especially during the COVID-19 pandemic and as a negative outcome of the lockdown [33]. Food insecurity is expected to be higher during these difficult times where the country is facing many financial, political, economic and health debates that put Lebanon in need of urgent policies to ensure food security, limiting by these the burden of the current situation on the Lebanese population, especially Lebanese children. The present study aims to find the prevalence of food insecurity among Lebanese children during the COVID-19 pandemic and its correlates such as the sociodemographic variables, lifestyle and eating habits.

## Methods

### Study design and participants

This cross-sectional study was carried out between March and April 2022. A total of 4001 parents was recruited from all Lebanese governorates. The research team contacted the Ministry of Education and Higher Education in Lebanon to disseminate the link to all parents of children aged between 5 and 11 years from public and private schools. Parents received an online link to the survey. They were encouraged to visit a website that would guide them to the consent form, information form (purpose of the current study, anonymity, voluntariness of consent to research), and questionnaire. All participants responded willingly to the survey. There were no fees for participating in the study. Excluded were those who refused to complete the survey.

### Minimal sample size calculation

According to the Epi-info software, a minimum of 1776 parents was deemed necessary to have enough statistical power, based on a 36% prevalence of food insecurity in Lebanon according to a previous study [34], 5% risk of error, and a design effect of 5.

### Questionnaire and variables

The lifestyle-related behavior during COVID 19 pandemic scale was forward translated to Arabic by one certified translator and then back translated to English by a second certified translator, following the international guidelines [35, 36].

The Arabic self-administered questionnaire with closed-ended questions was anonymous; the questionnaire required approximately 10 minutes to be completed. It consisted of different sections. The first part clarified socio-demographic characteristics: age, gender, marital status, education level, household monthly salary and household crowding index. The latter, reflecting the socioeconomic status of the family, was calculated by dividing the number of persons in the house by the number of rooms in the house excluding the bathrooms and kitchen [37].

In addition, the survey included 18 statements about the eating and lifestyle habits of children during the COVID-19 pandemic regarding skipping meals, eating snacks, eating legumes/fruits, sleeping habits, physical activity, etc. compared to before it. These questions were obtained from a previous published paper [38], but tailored to the children. Responses were rated from − 2 = significant decrease to 2 = significant increase. The Cronbach’s alpha value of those items was 0.93.

Validated in Arabic, the Food Security Scale was used to evaluate the level of food insecurity in the household [39]. It is composed of 7 statements, with 6 of them rated as a yes/no type of answer. Higher scores would indicate higher food insecurity. The total score yielded 3 categories as follows: scores between 0 and 1 were classified as being food secure, scores of 2–4 as moderate food insecurity, whereas scores of 5–7 as severe food insecurity. The Cronbach’s alpha value of the FSS scale was 0.86.

### Statistical analysis

The SPSS software version 25 was used for the statistical analysis. Cronbach’s alpha values were recorded for the scales used. Descriptive statistics included frequencies/percentages for categorical variables and means/standard deviations for continuous variables. The Chi-square test was used to compare categorical variables, whereas the Analysis of Variance (ANOVA) test was used to compare three means. Significance was set at *p* < 0.05.

## Results

### Characteristics of the participants

A total of 4001 participants accepted to enroll in this study. The mean age of the participants was 36.76 ± 6.60 years, with 85.1% females. The majority had a university level of education (51.9%), with a mean financial burden score of 8.01 ± 2.30. Other details are summarized in Table 1.

**Table 1:** Sociodemographic and other characteristics of the participants (*N* = 4001)

Variable	N (%)
Governorate	
Beirut	346 (8.6%)
Mount Lebanon	1340 (33.5%)
North	1733 (43.3%)
South	284 (7.1%)
Bekaa	298 (7.4%)
Altitude of the area of residence	
0–600 m	2701 (67.5%)
601–1000 m	957 (23.9%)
> 1000 m	343 (8.6%)
Gender	
Male	596 (14.9%)
Female	3405 (85.1%)
Marital status	
Married	3825 (95.6%)
Divorced	48 (1.2%)
Widowed	128 (3.2%)
Education level	
Primary	506 (12.6%)
Complementary	400 (10.0%)
Secondary	1017 (25.4%)
University	2078 (51.9%)
Employment status	
Unemployed	2008 (50.2%)
Full-time job	1040 (26.0%)
Part-time job	431 (10.8%)
Retired	48 (1.2%)
Self-employed	474 (11.8%)
Currently working / studying from home	
No	3090 (77.2%)
Yes	911 (22.8%)
Monthly salary income	
< 675,000 LBP	633 (15.8%)
675,000-1,200,000 LBP	1054 (26.3%)
1,200,000-2,400,000 LBP	766 (19.1%)
2,400,000-3,200,000 LBP	492 (12.3%)
3,200,000-5,000,000 LBP	526 (13.1%)
> 5,000,000 LBP	530 (13.2%)
Overall health of your children	
Bad	186 (4.6%)
Acceptable	1087 (27.2%)
Good	1366 (34.1%)
Very good	978 (24.4%)
Excellent	384 (9.6%)
	**Mean ± SD**
Age (in years)	36.76 ± 6.60
Household crowding index (persons/room)	1.44 ± 0.71
Total number of children	2.68 ± 1.13
Number of children (5–9 years)	1.66 ± 0.79
Financial burden	8.01± 2.30

### Lifestyle and eating habits of the children

A higher percentage of children had a decreased consumption of junk food, sugar-sweetened beverages, and a decrease in the participation in physical activities. In addition, around 25% of the children had a decrease in the number of hours of sleep and sleep quality. Other details about lifestyle and eating habits of the children are summarized in Table 2.

**Table 2:** Lifestyle and Eating Habits of the participants

During the COVID-19 pandemic…..	Increased	Same	Decreased
how has your child’s probability of skipping one of the main meals (breakfast/ lunch/dinner) changed?	1293 (32.3%)	1317 (32.9%)	1391 (34.8%)
how has your child’s habit of snacking between meals changed?	1751 (43.8%)	1044 (26.1%)	1206 (30.1%)
how has your child’s quantity/portions of meals and snacks changed?	1609 (40.2%)	1146 (28.6%)	1246 (31.1%)
how has your child’s daily intake of fruits and vegetables changed?	1247 (31.2%)	1336 (33.4%)	1418 (35.4%)
how has your child’s intake of a balanced diet (including health ingredients such as whole wheat, pulses, legumes, eggs, nuts, fruits and vegetables) changed?	1109 (27.7%)	1424 (35.6%)	1468 (36.7%)
how has your child’s consumption of junk food/fast food and fried food changed?	1053 (26.3%)	1016 (25.4%)	1932 (48.3%)
how has your child’s intake of sugar-sweetened beverages (carbonated soft drinks, sugar-sweetened juices) changed?	1106 (27.6%)	1149 (28.7%)	1746 (43.6%)
how has your child’s consumption of sweets/ candies/ chocolate changed?	1515 (37.9%)	1001 (25.0%)	1485 (37.1%)
how has your child’s consumption of unhealthy food when he/she is bored or stressed or upset changed?	1620 (40.5%)	1017 (25.4%)	1364 (34.1%)
how has your child’s intake of immunity-boosting foods (lemon, turmeric, garlic, citrus fruits and green leafy vegetables) in the diet changed?	1402 (35.0%)	1379 (34.5%)	1220 (30.5%)
how has your child’s intake of nutrition supplements to boost immunity changed?	1270 (31.7%)	1415 (35.4%)	1316 (32.9%)
how has your child’s support in eating healthy changed?	1419 (35.5%)	1406 (35.1%)	1176 (29.4%)
how has your child’s participation in physical activities changed?	1077 (26.9%)	1054 (26.3%)	1870 (46.7%)
how has your child’s participation in leisure and household chores changed?	1580 (39.5%)	1318 (32.9%)	1103 (27.6%)
how has your child’s sitting and screen time changed?	2432 (60.8%)	798 (19.9%)	771 (19.3%)
how have your child’s hours of sleep changed?	1535 (38.4%)	1459 (36.5%)	1007 (25.2%)
how has your child’s quality of sleep changed?	1338 (33.4%)	1620 (40.5%)	1043 (26.1%)
how have your child’s stress and anxiety level changed?	2154 (53.8%)	1011 (25.3%)	836 (20.9%)

### Food security

The mean FSS score was 3.48 ± 2.30. The results showed that 999 (25.0%) were food secure, 1505 (37.6%) had moderate food insecurity, whereas 1497 (37.4%) had severe food insecurity.

### Bivariate analysis of factors associated with food insecurity categories

A significantly higher percentage of families with severe food insecurity was reported by fathers compared to mothers. In addition, participants living in Bekaa, living in villages > 1000 m of altitude, with a primary level of education, retired, currently not working or studying from home, with a monthly salary < 675,000 LBP, and those who reported a bad overall health status of their children had a severe food insecurity. Moreover, those with a severe food insecurity had their children’s daily snacking habit between meals decreased, with a decreased quantity of meals, intake of vegetables/fruits, the intake of balanced diet, junk food, sugar-sweetened beverages, consumption of sweets/candies/chocolate, consumption of unhealthy food, intake of immunity-boosting food, intake of nutrition supplements, participation in the house chores, number of sleeping hours and sleep quality, as well as stress/anxiety decreased. Finally, a higher mean household crowding index, total number of children and financial burden was seen in families with severe food insecurity compared to the other groups (Table 3).

**Table 3:** Bivariate analysis of factors associated with the food insecurity categories

	Food secure	Moderate food insecurity	Severe food insecurity	***p***
Governorate				**< 0.001**
Beirut	130 (37.6%)	126 (36.4%)	90 (26.0%)	
Mount Lebanon	419 (31.3%)	576 (43.0%)	345 (25.7%)	
North	277 (16.0%)	589 (34.0%)	867 (50.0%)	
South	106 (37.3%)	101 (35.6%)	77 (27.1%)	
Bekaa	67 (22.5%)	113 (37.9%)	118 (39.6%)	
Altitude of the area of residence				**0.004**
0–600 m	711 (26.3%)	1029 (38.1%)	961 (35.6%)	
601–1000 m	218 (22.8%)	352 (36.8%)	387 (40.4%)	
> 1000 m	70 (20.4%)	124 (36.2%)	149 (43.4%)	
Gender of the parent				**< 0.001**
Male	105 (17.6%)	197 (33.1%)	294 (49.3%)	
Female	894 (26.3%)	1308 (38.4%)	1203 (35.3%)	
Marital status				0.267
Married	964 (25.2%)	1435 (37.5%)	1426 (37.3%)	
Divorced	8 (16.7%)	16 (33.3%)	24 (50.0%)	
Widowed	27 (21.1%)	54 (42.2%)	47 (36.7%)	
Education level				**< 0.001**
Primary	47 (9.3%)	123 (24.3%)	336 (66.4%)	
Complementary	52 (13.0%)	137 (34.3%)	211 (52.8%)	
Secondary	164 (16.1%)	388 (38.2%)	465 (45.7%)	
University	736 (35.4%)	857 (41.2%)	485 (23.3%)	
Employment status				**< 0.001**
Unemployed	381 (19.0%)	689 (34.3%)	938 (46.7%)	
Full-time job	325 (31.3%)	461 (44.3%)	254 (24.4%)	
Part-time job	97 (22.5%)	159 (36.9%)	175 (40.6%)	
Retired	6 (12.5%)	17 (35.4%)	25 (52.1%)	
Self-employed	190 (40.1%)	179 (37.8%)	105 (22.2%)	
Currently working / studying from home				**< 0.001**
No	728 (23.6%)	1123 (36.3%)	1239 (40.1%)	
Yes	271 (29.7%)	382 (41.9%)	258 (28.3%)	
Monthly salary income				**< 0.001**
< 675,000 LBP	59 (9.3%)	188 (29.7%)	386 (61.0%)	
675,000-1,200,000 LBP	122 (11.6%)	373 (35.4%)	559 (53.0%)	
1,200,000-2,400,000 LBP	106 (13.8%)	336 (43.9%)	324 (42.3%)	
2,400,000-3,200,000 LBP	113 (23.0%)	249 (50.6%)	130 (26.4%)	
3,200,000-5,000,000 LBP	231 (43.9%)	213 (40.5%)	82 (15.6%)	
> 5,000,000 LBP	368 (69.4%)	146 (27.5%)	16 (3.0%)	
Overall health of your children				**< 0.001**
Bad	15 (8.1%)	40 (21.5%)	131 (70.4%)	
Acceptable	137 (12.6%)	354 (32.6%)	596 (54.8%)	
Good	302 (22.1%)	569 (41.7%)	495 (36.2%)	
Very good	357 (36.5%)	415 (42.4%)	206 (21.1%)	
Excellent	188 (49.0%)	127 (33.1%)	69 (18.0%)	
**During the COVID-19 pandemic…..**				
how has your child’s probability of skipping one of the main meals (breakfast/ lunch/dinner) changed?				**< 0.001**
Increased	267 (20.6%)	515 (39.8%)	511 (39.5%)	
Remained the same	475 (36.1%)	509 (38.6%)	333 (25.3%)	
Decreased	257 (18.5%)	481 (34.6%)	653 (46.9%)	
how has your child’s habit of snacking between meals changed?				**< 0.001**
Increased	491 (28.0%)	700 (40.0%)	560 (32.0%)	
Remained the same	344 (33.0%)	395 (37.8%)	305 (29.2%)	
Decreased	164 (13.6%)	410 (34.0%)	632 (52.4%)	
how has your child’s quantity/portions of meals and snacks changed?				**< 0.001**
Increased	445 (27.7%)	643 (40.0%)	521 (32.4%)	
Remained the same	382 (33.3%)	461 (40.2%)	303 (26.4%)	
Decreased	172 (13.8%)	401 (32.2%)	673 (54.0%)	
how has your child’s daily intake of fruits and vegetables changed?				**< 0.001**
Increased	324 (26.0%)	500 (40.1%)	423 (33.9%)	
Remained the same	482 (36.1%)	532 (39.8%)	322 (24.1%)	
Decreased	193 (13.6%)	473 (33.4%)	752 (53.0%)	
how has your child’s intake of a balanced diet (including health ingredients such as whole wheat, pulses, legumes, eggs, nuts, fruits and vegetables) changed?				**< 0.001**
Increased	267 (24.1%)	432 (39.0%)	410 (37.0%)	
Remained the same	524 (36.8%)	578 (40.6%)	322 (22.6%)	
Decreased	208 (14.2%)	495 (33.7%)	765 (52.1%)	
how has your child’s consumption of junk food/fast food and fried food changed?				**< 0.001**
Increased	271 (25.7%)	419 (39.8%)	363 (34.5%)	
Remained the same	359 (35.3%)	387 (38.1%)	270 (26.6%)	
Decreased	369 (19.1%)	699 (36.2%)	864 (44.7%)	
how has your child’s intake of sugar-sweetened beverages (carbonated soft drinks, sugar-sweetened juices) changed?				**< 0.001**
Increased	263 (23.8%)	444 (40.1%)	399 (36.1%)	
Remained the same	416 (36.2%)	444 (38.6%)	289 (25.2%)	
Decreased	320 (18.3%)	617 (35.3%)	809 (46.3%)	
how has your child’s consumption of sweets/ candies/ chocolate changed?				**< 0.001**
Increased	441 (29.1%)	630 (41.6%)	444 (29.3%)	
Remained the same	350 (35.0%)	379 (37.9%)	272 (27.2%)	
Decreased	208 (14.0%)	496 (33.4%)	781 (52.6%)	
how has your child’s consumption of unhealthy food when he/she is bored or stressed or upset changed?				**< 0.001**
Increased	437 (27.0%)	664 (41.0%)	519 (32.0%)	
Remained the same	346 (34.0%)	402 (39.5%)	269 (26.5%)	
Decreased	216 (15.8%)	439 (32.2%)	709 (52.0%)	
how has your child’s intake of immunity-boosting foods (lemon, turmeric, garlic, citrus fruits and green leafy vegetables) in the diet changed?				**< 0.001**
Increased	370 (26.4%)	595 (42.4%)	437 (31.2%)	
Remained the same	451 (32.7%)	536 (38.9%)	392 (28.4%)	
Decreased	178 (14.6%)	374 (30.7%)	668 (54.8%)	
how has your child’s intake of nutrition supplements to boost immunity changed?				**< 0.001**
Increased	329 (25.9%)	516 (40.6%)	425 (33.5%)	
Remained the same	481 (34.0%)	565 (39.9%)	369 (26.1%)	
Decreased	189 (14.4%)	424 (32.2%)	703 (53.4%)	
how has your child’s support in eating healthy changed?				**< 0.001**
Increased	378 (26.6%)	586 (41.3%)	455 (32.1%)	
Remained the same	463 (32.9%)	543 (38.6%)	400 (28.4%)	
Decreased	158 (13.4%)	376 (32.0%)	642 (54.6%)	
how has your child’s participation in physical activities changed?				**0.004**
Increased	283 (26.3%)	392 (36.4%)	402 (37.3%)	
Remained the same	290 (27.5%)	412 (39.1%)	352 (33.4%)	
Decreased	426 (22.8%)	701 (37.5%)	743 (39.7%)	
how has your child’s participation in leisure and household chores changed?				**< 0.001**
Increased	414 (26.2%)	632 (40.0%)	534 (33.8%)	
Remained the same	393 (29.8%)	497 (37.7%)	428 (32.5%)	
Decreased	192 (17.4%)	376 (34.1%)	535 (48.5%)	
how has your child’s sitting and screen time changed?				**< 0.001**
Increased	693 (28.5%)	964 (39.6%)	775 (31.9%)	
Remained the same	193 (24.2%)	313 (39.2%)	292 (36.6%)	
Decreased	113 (14.7%)	228 (29.6%)	430 (55.8%)	
how have your child’s hours of sleep changed?				**< 0.001**
Increased	376 (24.5%)	611 (39.8%)	548 (35.7%)	
Remained the same	402 (27.6%)	573 (39.3%)	484 (33.2%)	
Decreased	221 (21.9%)	321 (31.9%)	465 (46.2%)	
how has your child’s quality of sleep changed?				**< 0.001**
Increased	317 (23.7%)	520 (38.9%)	501 (37.4%)	
Remained the same	462 (28.5%)	651 (40.2%)	507 (31.3%)	
Decreased	220 (21.1%)	334 (32.0%)	489 (46.9%)	
how have your child’s stress and anxiety level changed?				**< 0.001**
Increased	553 (25.7%)	853 (39.6%)	748 (34.7%)	
Remained the same	313 (31.0%)	388 (38.4%)	310 (30.7%)	
Decreased	133 (15.9%)	264 (31.6%)	439 (52.5%)	
Age of the parent	36.84 ± 6.12	36.85 ± 6.80	36.61 ± 6.70	0.328
Household crowding index	1.12 ± 0.48	1.35 ± 0.58	1.74 ± 0.82	**< 0.001**
Total number of children	2.44 ± 0.96	2.58 ± 1.08	2.94 ± 1.24	**< 0.001**
Number of children aged 5–10 years	1.54 ± 0.67	1.58 ± 0.72	1.82 ± 0.90	**< 0.001**
Financial burden	6.25 ± 2.63	8.08 ± 1.92	9.12 ± 1.61	**< 0.001**

Numbers in bold indicate significant *p*-values

## Discussion

Our results showed that parents with a monthly salary < 675,000 LBP (the minimum wage in Lebanon), with a higher mean household crowding index (lower socioeconomic status) and a high financial burden had severe food insecurity. Our results showed a significant increase in food insecurity rates to 37.6 and 37.4% of moderate and severe food insecurity rates respectively, compared to a previous study done in 2016 [34].

Our study results showed that fathers answering the survey revealed more severe food insecurity compared to mothers answering it, in opposite to a previous Lebanese research done in August 2021 showing the opposite finding [40]. In addition, our results showed that parents with a primary level of education and those unemployed/retired had more food insecurity, corroborating other studies that linked food insecurity with low educational levels, low monthly salary and unemployment [41–45]. This might be because banks are not allowing their clients to withdraw money from their own accounts, forcing them to live under the poverty line.

The results of this study also showed that children with severe food insecurity decreased skipping their meals during the pandemic with less consumption of vegetables/fruits, thus they were less susceptible to having a balanced healthy diet, even their intake of unhealthy food like junk food, sugary beverages, sweets, candies, chocolates decreased. They were more prone to take less daily snacks and they used to eat less immunity-boosting food. These findings are similar to other studies showing that food quality and quantity were reduced since their households are of lower socioeconomic status so they will only be able to pay for cheaper and less nutritious products [46, 47]. Food insecure children in this study had low consumption of junk food, which was not consistent with previous studies that showed a high fast food intake due to its lower costs compared to fresh healthy nutritious food like grains, fruits and vegetables [47–49]. Similar findings were observed in this study compared to other studies regarding lower intake of fruits and vegetables in food insecure children due to lower prices of frozen food and calorie-dense food [47, 49].

This study also showed lower consumption of sweets, sugary beverages, chocolates and unhealthy food in the food insecure group, which does not agree with the results of other studies since these are considered of low-cost and are more affordable to low-income families [50]. Decreased snacking habits were also found in food insecure children, in opposite to other studies that showed increased snacking in children during the pandemic [51, 52]. The low budget constraint and limited ability to buy sweets, sugary beverages, chocolates and fruits that are considered snacks can explain the results of this study. Food insecure children were shown not to skip their meals to meet their hunger, especially that they are not able to eat many snacks during their day.

In contrast, food insecure children in our study showed a decreased level of anxiety and stress, which was opposite to other studies reclaiming that food insecurity increase the risk of having psychological troubles in children disturbing their mental health such as anxiety, stress and depression [46, 53–55]. Children might face feelings of worry, shame and sadness [56, 57] regarding their reality and as a mechanism to cope with their chronic stress and may refer to bad quality diets to satisfy their hunger [50].

Moreover, this study showed that food insecurity had a negative impact on sleeping pattern in children with decreased sleeping hours and poor quality of sleep, which was consistent with other findings [58]. This might be considered a psychological impact of food insecurity on children [59, 60]. The results also showed that food insecurity was also linked with decreased physical activity in children, which can be explained by their decreased energy and physical weakness due to inadequate and insufficient healthy diet [61] and the lockdown restrictions (closure of sports fields, closure of public parks…).

The present study also showed that children spent more time on screens during the pandemic, in agreement with previous findings [62]. As a direct effect of the lockdown, children might face boredom and loneliness, thus refer to watching TV, playing video games and using the media to socialize and remain engaged in a locked-down world [63].

Lebanon had a lower rate of food insecurity of 5.2% and of an average 13.1% between 2015 and 2017 with an expectation to reach a rate of 20 to 24% between 2020 and 2022 at that time [34]. The results of this study went beyond these expectations, reaching an alarming rate of food insecurity in Lebanon during the pandemic. Lebanon had high levels of food security due to the availability of water, soil, adequate climate and diversity of Lebanese meals [64, 65]. The Lebanese economy relied for years on tourism, external money and investments [66] that decreased during the pandemic for security reasons in addition to the economic crisis and the Lebanese currency deterioration. Lebanon had weather changes causing a drop in its reserve of water, affecting negatively its agriculture [67]. The accumulation of economic and political instability, terrorism, the Beirut blast that caused the port destruction, the accumulation of debts from many wars, the protest since 2019, banking crisis and lack of medications threatened the food security and general health in Lebanon. During the pandemic, one-third of the Lebanese became unemployed whereas one-fifth noted significant reduction in their salaries especially with financial deterioration of the daily Lebanese Lira rates. Some had fears of lacking food, while others needed help to maintain their regular monthly income [68].

With the destruction of the Beirut port, the storage of food and imports were threatened, especially essential sources like grain. In addition, COVID-19 lockdown decreased the imports to the Lebanese population in general by 41.6% in 2020, especially food imports by 14.6% [69]. Lebanon is passing through a severe economic drop that dropped the Lebanese capacity of obtaining their human basic rights including healthcare facilities [70]. Higher prices of essential imported products like food and medications also played a role in the country’s inflation especially with the anarchic daily lira exchange rate turning from 1515 LBP for each US dollar to reaching more than 31,000 LBP in the black market in a short period, without any official control or plans. These factors might be, among others, responsible of the increased food insecurity rates in the country.

### Clinical implications

The results of this study are alarming and call for an urgent intervention to elaborate some policies and adopt strategies to assume food security. Lebanon depends on imports to afford food for Lebanese citizens even though the climate and water supply are favorable for variable agriculture. Plans to ameliorate and increase investments in this sector were disturbed because of the huge urbanization since the civil war. The Lebanese Ministry of Agriculture put a plan in 2020 trying to increase activities in this sector especially fresh fruits and vegetables in order to let the food supply meet the food demand. Nevertheless the lack of definitive strategy was the main reason behind the accomplishment of the project [71]. In addition, the deterioration of the Lebanese currency to the US dollar during the pandemic, on top of the sociopolitical and economic crises and the negative impact of COVID-19 lockdown by itself, made a huge increase in food prices, which affected food choices and the Lebanese population’s capacity to buy nutritious sufficient food [72, 73]. Lebanese people of low socioeconomic status note being dependent on food assistance to obtain their own food supply, which suggests that Lebanon needs more poverty targeting programs to help people in need. Food prices should be monitored to assume affordable prices to the whole population and avoid anarchic elevation of food prices. Moreover, shortage in food supply should be controlled especially healthy fresh food. The lack of supply contributed to panic behaviors among population, this is why food chains should be open and available to all Lebanese continents. Local farmers should be encouraged with protection programs to increase food supply even for those living with limited budgets thus decreasing by this the inflation rate.

### Limitations

An information bias is definitely present like in all observational studies, where participants tend to over- or underestimate the answer to a question. Although the method of data collection was random and at the national level, a selection bias is possible since we could not reach parents of children not enrolled in schools and because of the refusal rate (which could not have been assessed in this paper). The behavior lifestyle scale is not validated in Arabic in Lebanon. The questionnaire is self-administered, which can cause recollection and social desirability bias. The link was sent to all governorates; however, the response rate was fewer in some governorates compared to others. Despite these limitations, we believe that our results can be generalized to the whole population.

## Conclusion

The objectives of this study aimed at assessing the prevalence of food insecurity among the Lebanese population during the COVID-19 pandemic and the factors associated with it. The results showed a high prevalence of moderate to severe food insecurity among Lebanese children during the COVID-19 pandemic. Many factors made the Lebanese population susceptible to be food insecurity during the lockdown; these factors include the economic, political, financial crisis, inflation and deterioration of the Lebanese Lira. Food insecurity had a negative impact on children, leading to worse health status, eating habits, increase screen time, decrease quality of sleep and emotional distress. This issue should be seriously discussed in Lebanon due to its rapid development in the middle of all the crises facing the country in order to avoid short and long term consequences on human’s health. Serious strategies and interventions to prevent further inflation are needed in our country and should be discussed urgently with a clear objective to assume food security. Further studies are needed to find the possibility to implement strategies and adequate plans to encourage internal agriculture, taking into consideration the resources and capacities of the country. Targets might include decreasing food prices, food wasting and unemployment in addition to improvement of infrastructural programs, trade policies and diversification. Intervention is mandatory to avoid further hunger and impact of food insecurity on human’s physical and mental health.

## Data Availability

All the datasets used and/or analyzed during the current study available from the corresponding author (SH) on reasonable request.
